# 

**Published:** 1835-10-01

**Authors:** 


### CONTENTS

OF THE

### MEDICO-CHIRURGICAL REVIEW,

No. XLVI. OCTOBER 1, 1835.

[BEING No. VI. OF A DECENNIAL SERIES.]

### REVIEWS.

I.

A further Inquiry concerning Constitutional Irritation, and the Pathology of the Nervous System. By BENJAMIN TRAVERS, F.R.S... .. .. .. .. .. .. .. 297

II.

Clinique Medicale, &c.; or, select Cases treated in the CHARITE Hospital. By G. ANDRAL. Fifth Volume, "Diseases of the Encephalon." .. .. .. .. .. .. 345

1. Cerebral Hæmorrhage .. .. .. .. .. .. .. .. .. .. .. .. 346

2. Lesions of Motion.. .. .. .. .. .. .. .. .. .. .. .. .. 350

3. Lesions of Sensibility .. .. .. .. .. .. .. .. .. .. .. .. 352

4. Lesions of the Sensibility of the Mucous Membranes.. .. .. .. .. .. 354

5. Lesions of the Organs of Sense .. .. .. .. .. .. .. .. .. .. 355

6. Lesions of Intelligence .. .. .. .. .. .. .. .. .. .. .. . 357

7. Lesions of Nutrition .. .. .. .. .. .. .. .. .. .. .. .. 359

III.

The Constitution of Man, considered in relation to External Objects. By GEORGE COMBE, Esq.. . .. .. .. .. .. .. .. .. .. .. .. .. .. 361

IV.

A Treatise on Head-aches, their various Causes, Prevention, and Cure. By GEORGE HUME WEATHERHEAD, M.D. .. .. .. .. .. .. .. .. .. .. .. .. 370

V.

Outlines of Comparative Anatomy. By Dr. GRANT . .. .. .. .. .. .. .. 376

VI.

Observations on the Causes and Treatment of Ulcerous Diseases of the Leg. By J. C. SPENDER, Esq. .. .. .. .. .. .. .. .. .. .. .. .. .. .. 388

VII.

On the Power, Wisdom, and Goodness of God, as manifested in the Creation of Animals, and in their History, Habits, and Instincts. By the Rev. WILLIAM KIRBY, M.A. .. .. .. .. .. .. .. . .. .. .. .. .. .. 400

VIII.

CUTANEOUS DISEASES.—(Eclectic Article.)

1. A Theoretical and Practical Treatise on Diseases of the Skin. By P. RAYER, M.D.
2. Atlas of 26 coloured Engravings .. .. .. .. .. .. .. .. . . .. ..
3. A practical Compendium of the Diseases of the Skin. By JONATHAN GREEN ..
4. Cyclopædia of Practical Medicine .. .. .. .. .. .. .. .. .. ..

} 414

### PERISCOPE.

### PART I.

### Spirit of the English Periodicals, and Notices of English Medical Literature.

1. Cases of Carditis, with Remarks. By Dr. Stroud .. 441
2. Fatal Dissection Wound. By Dr. Benson.. .. 445
3. Dr. Graves on Dropsy .. .. 446
4. On the Physical and Chemical Properties, Preparation, Physiological Action, &c. &c. of Kreosote .. .. 448
5. On Occlusion of the Biliary Ducts, with Cases in Illustration.. .. 452
6. Observations on the Obstetric Extractor, usually called the Lever. By Dr. John Breen .. .. 454
7. Observations on Lepra and Psoriasis; with numerous Cases. By Dr. C.W. Pennock 457
8. On Erythema Nodosum. By Mr. Blackley .. .. 466
9. On the Cause of Sleep. By Mr. Carmichael .. .. 470
10. Case of Tracheitis, with the Operation of Tracheotomy, Dissection, &c. .. 472
11. Remarkable Case of Occlusion of the Vagina. By Dr. Hoillemin .. .. 474
12. Melancholy Effects of inordinate Depletion. By Professor Badham, with Strictures on the Case .. .. 475
13. On the Employment of the Stethoscope in the Diagnosis of Aneurisms of the Thoracic Aorta. By Dr. G. Greene, with numerous Cases .. .. 477
14. Successful Case of Traumatic Tetanus. By Mr. Grant .. .. 484
15. On the Exhibition of Tartarized Antimony in Tetanus. By Mr. Woodward.. .. 485
16. Dr. Hudson on Typhoid Pneumonia .. .. 485
17. A Series of Plates, illustrating the Causes of Displacement in the various Fractures of the Bones of the Extremities. By Mr. G. W. Hind .. .. 487

### British Association.

18. Dr. Graves on Chloride of Sodium in Fever .. .. 489
19. Dr. Houston on the Circulating Organs of Animals accustomed to Diving .. .. 490
20. Report of the Committee appointed to experiment on the Sounds and Motions of the Heart .. .. 491
21. Dr. M'Donnell on the Action of the Heart and the Pulse in Different Positions of the Body .. .. 493
22. Dr. Harrison on certain Bones found in the Hearts of some of the Ruminantia .. 495
23. Dr. Houston on Hydatids in the Omentum of the Axis Deer .. .. 496
Dr. Harrison on Entozoa in Muscles .. .. 496
Mr. Crampton on Worms found in the Tracheæ of Deer .. .. 497
24. Dr. Alison on the Vital Properties of Arteries leading to Inflamed Parts.. .. 497
25. Mr. Whatton on Partial Amputation of the Foot .. .. 499
26. Dr. Stokes on the Diagnosis of some Diseases of the Thorax .. .. 501
27. Dr. Kennedy on the Treatment of Purulent Ophthalmia in Infants.. .. 503
28. Dr. Beatty's Remarks on Ophthalmia.. .. 505
29. Case of Cæsarean Operation, successfully performed by Mr. Knowles .. .. 505
30. Dr. Corrigan on the Bruit de Soufflet.. .. 505
31. Dr. O'Beirne on Defæcation.. .. 508
32. Dr. Osborne on the Effects of Cold and Climate.. .. 508
33. Dr. Hutton on Congenital Defects .. .. 511
34. Mr. Adams on Aneurism by Anastomosis .. .. 511

35. Mr. Acret on the Varieties of Trusses (Review) .. .. .. .. .. .. .. 512
36. Dr. W. Philip's Gulstonean Lectures—Obscure Affections of the Brain (Review) 515
37. Dr. Elliotson on Subcutaneous Inflammation, and Nitrate of Silver .. .. .. 519

### PART III.

### Clinical Review and Hospital Reports.

PENNSYLVANIA HOSPITAL.

38. Report of Cases Treated in the Surgical Wards. By Dr. T. S. Kirkbride.
  1. Hernia, Cases of .. .. .. .. .. .. .. .. .. .. .. .. .. .. 521
  2. Tardy Union and Absorption of Callus .. .. .. .. .. .. .. .. .. 523
  3. Dislocation of Os Humeri on the Dorsum of the Scapula .. .. .. .. 525
  4. Injuries of the Abdomen .. .. .. .. .. .. .. .. .. .. .. .. 526
  5. Sloughing about the Anus. Kreosote .. .. .. .. .. .. .. .. 527
  6. Temporary Loss of Vision .. .. .. .. .. .. .. .. .. .. .. 528

SIR P. DUN'S HOSPITAL.

39. Recovery from a State of Fever almost desperate. Dr. Graves .. .. .. .. 529

BRISTOL INFIRMARY.

40. On Osteo-Sarcoma of both Jaws, with a formidable Operation, and Practical Remarks. By W. Hetling, Esq. (Review) .. .. .. .. .. .. .. .. 531

GUY'S HOSPITAL.

41. Mr. Anderson on Renal Dropsy .. .. .. .. .. .. .. .. .. .. .. 537

NORTH LONDON HOSPITAL.

42. Employment of Kreosote for the Prevention of Sickness in various Diseases. By Mr. Taylor .. .. .. .. .. .. .. .. .. .. .. .. .. .. 541

MANCHESTER ROYAL INFIRMARY.

43. Clinical Report on Diabetes. By Dr. Carbutt (Review) .. .. .. .. .. .. 542

MARYLEBONE INFIRMARY.

44. Dr. Clendinning's Statistical Report from that Institution .. .. .. .. .. 549

GLASGOW INFIRMARY.

45. Dr. M'Farlane's Report of various Cases of Hernia .. .. .. .. .. .. 551

ST. GEORGE'S HOSPITAL.

46. Dr. M'Leod's Report of Cases .. .. .. .. .. .. .. .. .. .. .. 558

ROYAL INFIRMARY OF EDINBURGH.

47. Mr. Syme's Report of Surgical Cases for the Winter Session .. .. .. .. .. 560

MERCER'S HOSPITAL.

48. Dr. Osborne on Diseases of the Stomach .. .. .. .. .. .. .. .. .. 562

LOCK HOSPITAL.

49. On Syphilitic Sores in the Urethra. By Mr. Henry J. Johnson .. .. .. .. 564

EDINBURGH EYE INFIRMARY.

50. Mr. Watson's Report of Ophthalmic Cases .. .. .. .. .. .. .. .. 566
  1. Injuries of the Eye .. .. .. .. .. .. .. .. .. .. .. .. 567
  2. Iritis .. .. .. .. .. .. .. .. .. .. .. .. .. .. 567
  3. Cataract .. .. .. .. .. .. .. .. .. .. .. .. .. 568
51. The Hospitals and their Regulations .. .. .. .. .. .. .. .. .. .. 569

### Miscellanies.

52. Dr. Madden's Residence in the West Indies .. .. .. .. .. .. .. .. 574

  1. Yellow Fever .. .. .. .. .. .. .. .. .. .. .. .. .. 574

  2. Sangaree .. .. .. .. .. .. .. .. .. .. .. .. .. 575

  3. The Mosquitoes .. .. .. .. .. .. .. .. .. .. .. .. 575

53. Philanthropic Economy .. .. .. .. .. .. .. .. .. .. .. .. .. .. .. 576
54. Quain's Anatomical Plates .. .. .. .. .. .. .. .. .. .. .. .. .. .. 576
55. The Harveian Oration. By Sir Henry Halford, Bart... .. .. .. .. .. .. 577
Obituaries of Tuthill, Maton, Powell.. .. .. .. .. .. .. .. .. .. .. .. 577
56. Atlas of Cutaneous Diseases .. .. .. .. .. .. .. .. .. .. .. .. .. .. 578
57. Banffshire Medico-Chirurgical Society .. .. .. .. .. .. .. .. .. .. .. 579
58. Obituary Extraordinary—Life and Death of the Quarterly Medical Review .. 579
59. Bibliographical Record .... . .. .. .. .. .. .. .. .. .. .. .. 583

